# Increased tortuosity of ACA might be associated with increased risk of ACoA aneurysm development and less aneurysm dome size: a computer-aided analysis

**DOI:** 10.1007/s00330-019-06146-3

**Published:** 2019-04-11

**Authors:** Roger M. Krzyżewski, Kornelia M. Kliś, Borys M. Kwinta, Małgorzata Gackowska, Jerzy Gąsowski

**Affiliations:** 1 Department of Neurosurgery and Neurotraumatology, Jagiellonian University Medical College, Botaniczna 3 Street, 31-503 Kraków, Poland; 2 TENSOR- Team of NeuroSurgery-Oriented Research, Jagiellonian University Medical College, Kraków, Poland; 3 Faculty of Medicine, Jagiellonian University Medical College, Kraków, Poland; 4 Faculty of Computer Science, Electronics and Telecommunications, AGH University of Science and Technology, Kraków, Poland; 5 Department of Internal Medicine and Gerontology, Jagiellonian University Medical College, Kraków, Poland

**Keywords:** Anterior communicating artery aneurysm, Risk factors, Anterior cerebral artery

## Abstract

**Objectives:**

We decided to perform computer-aided analysis of the anterior cerebral artery (ACA) to check for a potential correlation with anterior communicating artery (ACoA) aneurysm presence and growth.

**Methods:**

We retrospectively analyzed the ACA anatomy of 121 patients with ACoA aneurysms along with 121 age, risk factors, and vessel side-matched control patients without an ACoA aneurysm. We obtained their medical history and digital subtraction angiography (DSA) data from their medical records. For each patient’s DSA, we extracted curve representing the course of their ACA and calculated its relative length (RL), sum of angle metrics (SOAM), triangular index (TI), product of angle distance (PAD), and inflection count metrics (ICM).

**Results:**

Patients with ACoA aneurysm had significantly higher RL (0.64 ± 0.23 vs. 0.56 ± 0.22; *p* < 0.01), SOAM (0.27 ± 0.19 vs. 0.18 ± 0.15; *p* < 0.01), PAD (0.12 ± 0.13 vs. 0.09 ± 0.11; *p* = 0.02), and TI (0.57 ± 0.14 vs. 0.44 ± 0.15; *p* < 0.01). In multivariate logistic regression analysis, after adjustment for possible confounders, SOAM (OR, 1.34; 95% CI, 1.12–1.63; *p* < 0.01) and TI (OR, 1.84; 95% CI, 1.47–2.35; *p* < 0.01) remained independently associated with higher risk of ACoA aneurysm. Additionally, we found significant negative correlations between TI and aneurysm dome size (*R* = − 0.194; *p* = 0.047).

**Conclusions:**

Increased tortuosity of ACA might increase the risk of ACoA aneurysm development and decrease the risk of aneurysm growth.

**Key Points:**

*• Anterior cerebral artery’s sum of angle metrics is associated with hypertension as well as with history of ischemic stroke and myocardial infarction.*

*• Increased tortuosity of anterior cerebral artery might be associated with anterior communicating artery aneurysm development.*

*• Tortuosity of anterior cerebral artery is negatively correlated with anterior communicating artery aneurysm dome size.*

## Introduction

Vessel tortuosity has been found in a number of organ systems [1], is associated with several systemic diseases, such as hypertension and diabetes mellitus [2, 3], and may be indicative of vascular pathologies [4, 5] and increases with age [6]. Tortuosity has hitherto been analyzed mostly in terms of retinal and coronary vessel anatomy, although it also occurs in cerebral vessels, both in larger arteries and white matter arterioles [1, 7]. Cerebral vessel tortuosity has been previously documented to coexist with hypertension and moyamoya disease [8, 9]. Additionally, brain tumor vasculature tortuosity was analyzed as a potential predictor of its malignancy [10]. The mechanisms that can induce arterial tortuosity include mechanical factors of blood flow, such as increased blood pressure [2], reduced axial tension, and artery elongation [1], and arterial wall weakening, resulting from elastin degradation or abnormal deposits within the vessel wall [11]. Tortuosity could also result from degradation of surrounding tissue [12]. The association of tortuosity with those phenomena might imply that higher tortuosity promotes the development of such vascular pathologies as intracranial aneurysms. To the best of our knowledge, tortuosity of only two cerebral arteries—internal carotid artery and vertebral artery—has thus far been shown to be associated with higher risk of intracerebral aneurysm development [13, 14]. Previously, we were the first ones to demonstrate the relationship between aneurysm formation and tortuosity of the middle cerebral artery (MCA) [15]. However, no association between anterior cerebral artery (ACA) tortuosity and anterior communicating artery (ACoA) aneurysm development has thus far been reported.

We hypothesize that ACA tortuosity might be related to the risk of ACoA aneurysm formation and therefore we performed computer-aided analysis of ACA tortuosity and its relation with the presence, type, and propensity for progression of ACoA aneurysms.

## Materials and methods

### Patients

We retrospectively analyzed data of patients with intracranial aneurysm, confirmed by digital subtraction angiography (DSA), hospitalized between January 2013 and November 2017, at the Department of Neurosurgery, Jagiellonian University, University Hospital, Kraków, Poland. Patients who underwent clipping or coiling of the aneurysm were excluded from our study. We selected 121 patients with ACoA aneurysms. The control group consisted of 121 patients without an intracranial aneurysm or with aneurysm located on different arteries than ACoA, ACA, or internal carotid artery (ICA). We aimed to match the control group for age (± 2 years), side of ACA, and risk factors including hypertension, diabetes mellitus, and smoking. From patients’ medical records, we obtained their medical history, including previous and current diseases and medications. Patients with multiple aneurysms and connective tissue disorders were excluded from our study. The study protocol was approved by the Ethics Committee of the Jagiellonian University and written consent was obtained from each patient included in the study. The study protocol conforms to the ethical guidelines of the 1975 Declaration of Helsinki. The data that support the findings of this study are available from the corresponding author upon reasonable request.

### Software

To perform all image transformations and factor calculations, we used original software written by the first author of this study in C# 7.0 using Microsoft Visual Studio Community 2017 (Microsoft Corporation) licensed for non-commercial use together with Emgu CV image processing library.

### Image processing

**Fig. 1:**
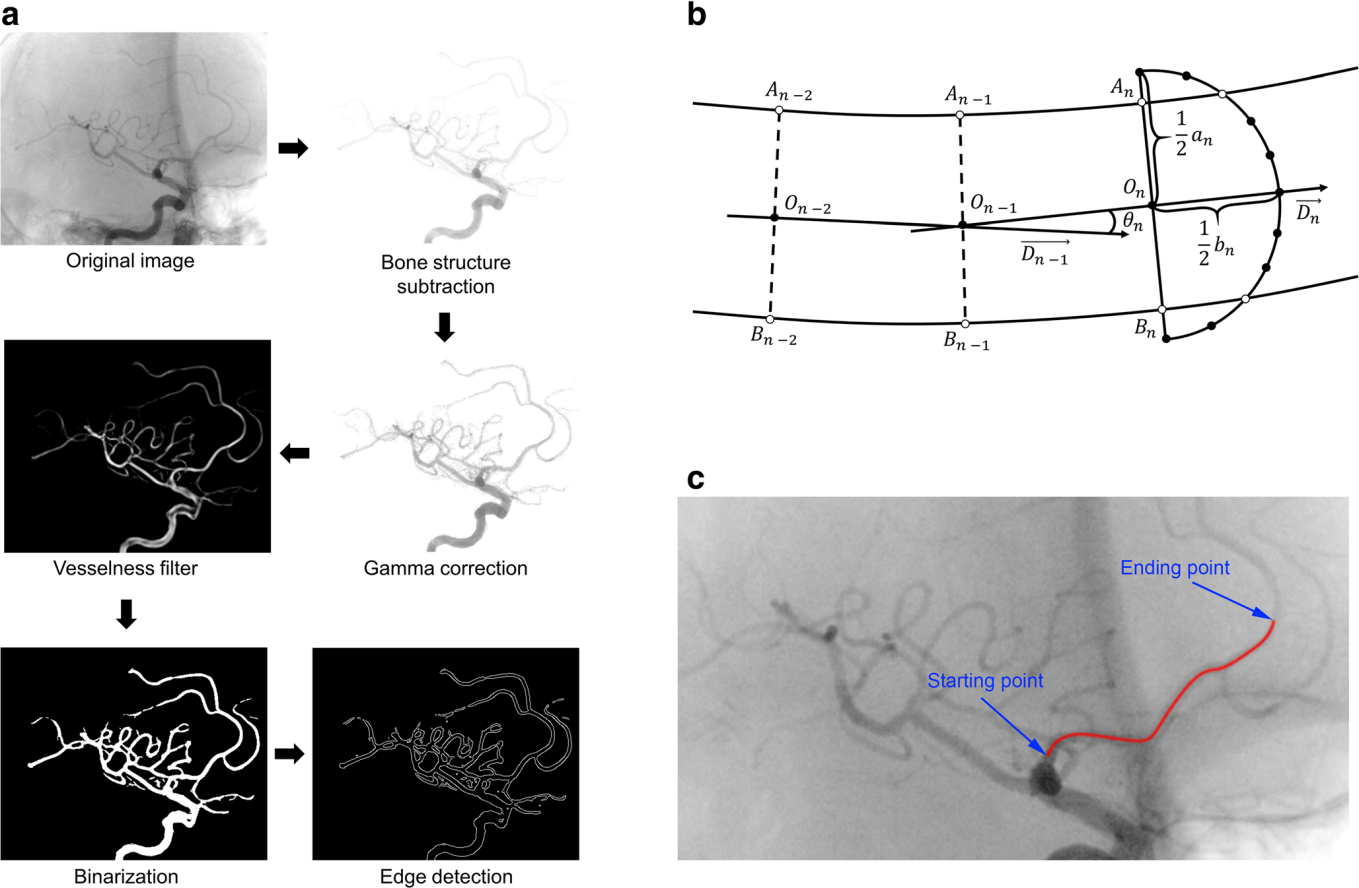
The anterior cerebral artery tracking process. **a** Image transformations of each patient’s digital subtraction angiography leading to vessel edge detection. **b** Schema of vessel edge point detection. *O*_*n*_ – searching region center, *a*_*n*_ – searching region major axis length, *b*_*n*_ – searching region minor axis length, $\overrightarrow{D_n}$ – direction vector, *θ*_*n*_ – angle between $\overrightarrow{D_n}$ and $\overrightarrow{D_{n-1}}$, A, B – detected edge point sets. **c** Final result of anterior cerebral artery tracking between selected starting and ending points

To detect ACA course, we performed a series of image transformation on the lateral projection of each patient’s DSA. First, the bone structures were subtracted and we performed gamma correction to increase visibility of blood vessels. Then, we applied multiscale vessel enhancement filter [16] to detect all vessel-like structures on the image. After that, images were binarized and we used the Canny edge detector to find vessel edges (Fig. 1a).

### ACA tracking

We analyzed A1, A2, and A3 segments of the ACA. In the first step, starting and ending point of the tracking were manually selected. Then, their nearest edge points were identified. Searching for other points was performed stepwise along the vessel axis: in the *n*th step, semi-ellipse shape-searching region was defined with center *O*_*n*_, major axis length *a*_*n*_, minor and axis length *b*_*n*_, and a direction vector $\overrightarrow{D_n}$. *O*_*n*_ was defined as the midpoint between previously found edge points, $\overrightarrow{D_n}$ was defined as $\overrightarrow{O_{n-1}\ {O}_n}$, and ellipse axes were calculated with the given formulas:

$$ {\displaystyle \begin{array}{c}{a}_n=2{d}_n\\ {}{b}_n=\frac{1\kern1em -{\theta}_n}{\pi }\ {d}_n\end{array}} $$

where *d*_*n*_ is the distance between two previously selected points and *θ*_*n*_ is the angle between $\overrightarrow{D_n}$ and $\overrightarrow{D_{n-1}}$. Finding of more or less than two edge points in the *n*th step was treated as vessel crossing or branching, and points located in that step were not taken into consideration. Curve-connecting midpoints between each pair of edge points, representing the ACA course, were used for further analysis (Fig. 1).

### Relative length

**Fig. 2:**
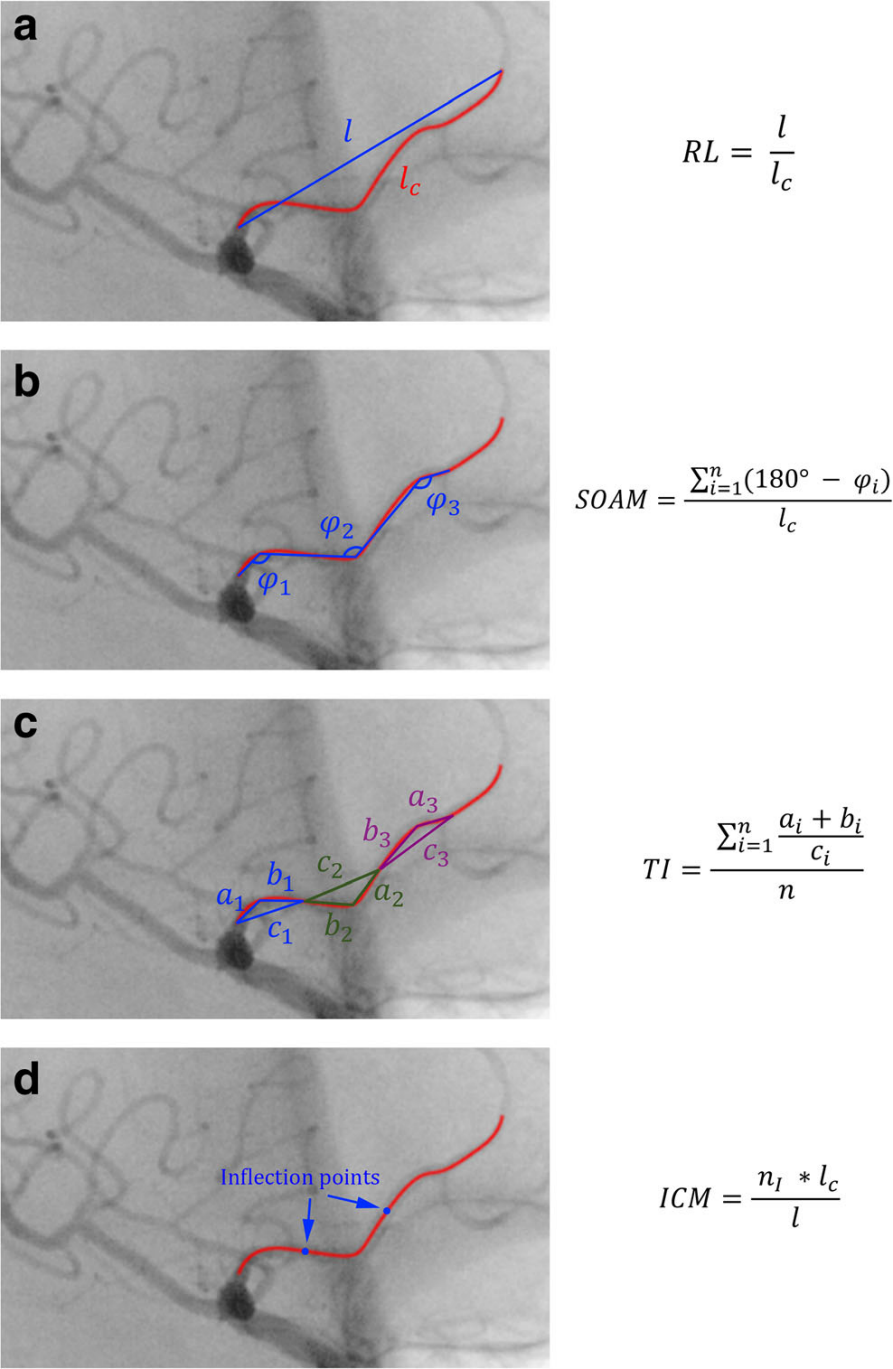
The tortuosity descriptor calculation. **a** RL – relative length. **b** SOAM – sum of angle metrics, *n* – number of measured angles, *l*_*c*_ – curve length. **c** TI – triangular index, *n* – number of constructed triangles. **d** ICM – inflection count metrics, *n*_*I*_ – number of inflection points, *l*_*c*_ – curve length, *l* – distance between starting and ending points of curve. *Reprinted from Krzyżewski RM, Kliś KM, Kwinta BM,* et al *Analysis of anterior cerebral artery tortuosity: association with anterior communicating artery aneurysm rupture. World Neurosurg. 2018, doi:*10.1016/j.wneu.2018.10.086*, with permission from Elsevier*

The first of used tortuosity descriptors is RL, calculated with the given formula:

$$ RL=\frac{l}{l_c} $$

where *l*_*c*_ is the curve length and *l* is the length of the straight line between the start and end points of the curve. RL is lower for curves which are more deviated from straightness (Fig. 2a).

### Sum of angle metrics

Calculation of SOAM begins from the division of curve into the subcurves of equal length. Then, for each subcurve, we calculate supplementary of the angle between lines connecting its center and ends. Angles are summed up and the result is normalized by the length of the entire curve:

$$ \mathrm{SOAM}=\frac{\ {\sum}_{i=1}^n\left({180}^{{}^{\circ}}-{\varphi}_i\right)}{l_c} $$

where *φ* indicates measured angles and *n* is its count. For a curve that is straighter, supplementary angles are larger and therefore the entire SOAM is larger. This descriptor is also sensitive to local tortuosity (Fig. 2b).

### Product of angle distance

The PAD is calculated using the below formula:

$$ PAD=\frac{SOAM}{RL} $$

This descriptor defines global tortuosity which is the combination of deviation from the straight line given by RL and local angles metrics, which is given by the SOAM.

### Triangular index

To calculate TI, the curve must be again divided into equal subcurves. Then, a triangle is built with vertices on each end of the subcurve and in its middle point. TI is calculated using the below formula:

$$ \mathrm{TI}=\frac{\ {\sum}_{i=1}^n\frac{a_i+{b}_i}{c_i}}{n} $$

where *n* is the number of subcurves, *a* and *b* are the triangle sides, and *c* is its base. TI is smaller for more straight curves (Fig. 2c).

### Inflection count metrics

Inflection point is a point on a curve where it changes from being concave to being convex, or vice versa. ICM is defined with below formula:

$$ \mathrm{ICM}=\frac{\ {n}_I\times {l}_c}{l} $$

where *n*_*I*_ is the number of curve’s inflection points. That factor is more sensitive to global curvature than RL and is lower for more straight curves (Fig. 2d).

### Additional measurements

For each analyzed segment of ACA, we measured its diameter, which was defined as the shortest distance between the artery edge points in the selected region. In a similar way, we measured the diameter of C7 segment of ICA. We also obtained the length of A1–A3 ACA segments.

### Statistical analysis

The database management and statistical analysis were performed with RStudio version 8.5 for Windows (RStudio, Inc).

We used the Shapiro-Wilk test to assess normality. For comparisons of continuous variables, we used the *t* test for normally distributed variables and the Mann-Whitney *U* test for non-normally distributed variables. We used the *χ*^2^ test for the dichotomized variables. To assess the correlation between continuous variables, we used Pearson’s or Spearman’s correlation tests, for normally and non-normally distributed variables, respectively. We express continuous variables as mean ± standard deviation. To find factors independently associated with the presence of the ACoA aneurysm, we employed logistic regression analysis, with and without adjustment for possible confounders. All significance tests are two-tailed and the *p* value of < 0.05 has been considered significant.

## Results

### Study group characteristics

The mean age of 242 (62.40% female) patients was 59.2 ± 12.6 years. The indices of ACA anatomy were as follows: the mean diameter of A1 segment was 1.77 ± 0.44 mm, mean diameter of A2 segment was 1.47 ± 0.43 mm, mean diameter of A3 segment was 1.28 ± 0.38 mm, and mean length of A1–A3 segments was 10.27 ± 2.17 mm. The average diameter of ICA C7 segment was 3.47 ± 0.78 mm. For A1–A3 ACA segments, mean RL was 0.60 ± 0.23, mean SOAM was 0.23 ± 0.18, mean PAD was 0.11 ± 0.12, mean TI was 0.50 ± 0.16, and mean ICM was 0.09 ± 0.12. Among patients with ACoA aneurysm, average dome size was 4.95 ± 2.82 mm. Among patients in the control group, 72 (59.02%) had intracranial aneurysm. The most common location of these aneurysms was the middle cerebral artery (54.16%), then the basilar artery (29.17%), posterior communicating artery (13.88%), and vertebral artery (2.78%).

### Association of risk factors with tortuosity

SOAM was lower in women than in men (0.20 ± 0.16 vs. 0.27 ± 0.20; *p* = 0.01), as was PAD (0.09 ± 0.13 vs. 0.13 ± 0.12; *p* = 0.04) and TI (0.48 ± 0.16 vs. 0.54 ± 0.16; *p* < 0.01). Additionally, patients with hypertension had significantly higher SOAM (0.25 ± 0.17 vs. 0.20 ± 0.19; *p* = 0.04). We found no significant differences in tortuosity indices according to the presence or absence of diabetes mellitus, hypercholesterolemia, or active smoking. However, patients with a history of myocardial infarction had significantly higher SOAM (0.44 ± 0.23 vs. 0.22 ± 0.17; *p* < 0.01). Similarly, SOAM was significantly higher among patients with a history of ischemic stroke (0.28 ± 0.20 vs. 0.21 ± 0.17; *p* = 0.02) (Table 1). We found no significant correlation between age and indices of tortuosity.

**Table 1:** Association of intracerebral aneurysm development risk factors with anterior cerebral artery tortuosity descriptors

	Female gender (*n* = 151)	Male gender (*n* = 91)	*p* value
Relative length	0.61 ± 0.22	0.60 ± 0.23	0.76
Sum of angle metrics	0.20 ± 0.16	0.27 ± 0.20	< 0.01
Product of angle distance	0.09 ± 0.13	0.13 ± 0.12	0.04
Triangular index	0.48 ± 0.16	0.54 ± 0.16	< 0.01
Inflection count metric	0.09 ± 0.11	0.10 ± 0.14	0.37
	Hypertension (*n* = 116)	No hypertension (*n* = 126)	
Relative length	0.58 ± 0.21	0.62 ± 0.24	0.22
Sum of angle metrics	0.25 ± 0.17	0.20 ± 0.19	0.04
Product of angle distance	0.11 ± 0.10	0.10 ± 0.14	0.54
Triangular index	0.51 ± 0.15	0.50 ± 0.18	0.40
Inflection count metric	0.08 ± 0.08	0.10 ± 0.15	0.23
	Diabetes mellitus (*n* = 35)	No diabetes mellitus (*n* = 207)	
Relative length	0.59 ± 0.23	0.60 ± 0.23	0.68
Sum of angle metrics	0.25 ± 0.16	0.22 ± 0.18	0.47
Product of angle distance	0.12 ± 0.15	0.10 ± 0.12	0.44
Triangular index	0.47 ± 0.17	0.51 ± 0.16	0.21
Inflection count metric	0.09 ± 0.15	0.09 ± 0.12	0.89
	Smoking (*n* = 42)	No smoking (*n* = 200)	
Relative length	0.61 ± 0.23	0.60 ± 0.23	0.89
Sum of angle metrics	0.20 ± 0.15	0.23 ± 0.18	0.37
Product of angle distance	0.10 ± 0.11	0.11 ± 0.13	0.66
Triangular index	0.53 ± 0.17	0.50 ± 0.16	0.17
Inflection count metric	0.11 ± 0.17	0.09 ± 0.11	0.18
	Hypercholesterolemia (*n* = 23)	No hypercholesterolemia (*n* = 219)	
Relative length	0.53 ± 0.20	0.61 ± 0.23	0.11
Sum of angle metrics	0.29 ± 0.16	0.22 ± 0.18	0.10
Product of angle distance	0.13 ± 0.08	0.10 ± 0.13	0.23
Triangular index	0.51 ± 0.14	0.50 ± 0.17	0.73
Inflection count metric	0.11 ± 0.09	0.09 ± 0.13	0.52
	History of heart attack (*n* = 6)	No history of heart attack (*n* = 236)	
Relative length	0.61 ± 0.13	0.60 ± 0.23	0.94
Sum of angle metrics	0.44 ± 0.23	0.22 ± 0.17	< 0.01
Product of angle distance	0.16 ± 0.08	0.10 ± 0.13	0.23
Triangular index	0.58 ± 0.14	0.50 ± 0.16	0.26
Inflection count metric	0.11 ± 0.09	0.09 ± 0.12	0.71
	History of ischemic stroke (*n* = 49)	No history of ischemic stroke (*n* = 193)	
Relative length	0.60 ± 0.24	0.60 ± 0.22	0.95
Sum of angle metrics	0.28 ± 0.20	0.21 ± 0.17	0.02
Product of angle distance	0.13 ± 0.12	0.10 ± 0.13	0.13
Triangular index	0.53 ± 0.16	0.50 ± 0.17	0.22
Inflection count metric	0.12 ± 0.18	0.08 ± 0.1	0.06

### Factors associated with the presence of aneurysms

**Fig. 3:**
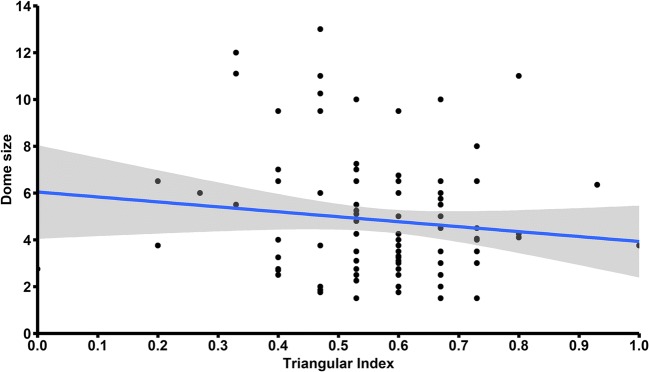
Scatter plot relating anterior cerebral artery triangular index against anterior communicating artery aneurysm dome size. Blue line indicates fitted linear regression line with confidence interval

Patients with ACoA aneurysm more often were male (76.9% vs. 47.9%; *p* < 0.01), less often took angiotensin-converting enzyme inhibitors (ACEI) (9.9% vs. 20.7%; *p* = 0.02), and more often took anticoagulants (8.3% vs. 0.80%; *p* < 0.01). In terms of artery sizes, patients with ACoA aneurysm had significantly longer A1–A3 ACA segments (10.70 ± 2.51 vs. 9.85 ± 1.68; *p* < 0.01) and significantly thinner A3 segment (1.20 ± 0.36 vs. 1.36 ± 0.38 mm; *p* < 0.01). They also had significantly higher RL (0.64 ± 0.23 vs. 0.56 ± 0.22; *p* < 0.01), SOAM (0.27 ± 0.19 vs. 0.18 ± 0.15; *p* < 0.01), PAD (0.12 ± 0.13 vs. 0.09 ± 0.11; *p* = 0.02), and TI (0.57 ± 0.14 vs. 0.44 ± 0.15; *p* < 0.01). We found no significant difference in terms of ICM between patients with and without ACoA aneurysm (0.10 ± 0.14 vs. 0.08 ± 0.09; *p* = 0.11) (Table 2). We found a significant negative correlation between TI and size of aneurysm dome size (*R* = − 0.19; *p* = 0.047) (Table 3, Fig. 3). In the multivariate logistic regression analysis, after adjustment for possible confounders, SOAM (OR, 1.34; 95% CI, 1.12–1.63; *p* < 0.01) and TI (OR, 1.84; 95% CI, 1.47–2.35; *p* < 0.01) remained independently associated with higher risk of ACoA aneurysm and female gender (OR, 0.33; 95% CI, 0.17–0.60; *p* < 0.01) remained independently associated with lower risk of ACoA aneurysm.

**Table 2:** Comparison of aneurysm development risk factors between the study and control group

	ACoA aneurysm (*n* = 121)	No ACoA aneurysm (*n* = 121)	*p* value
Female gender (%)	47.93 (58)	76.86 (93)	< 0.01
Age, years ± SD	60.22 ± 14.07	58.23 ± 10.95	0.22
Risk factors			
Diabetes mellitus (%)	13.22 (16)	15.7 (19)	0.58
Smoking (%)	16.53 (20)	18.18 (22)	0.73
Hypertension (%)	44.63 (54)	51.24 (62)	0.30
Alcoholism (%)	3.31 (4)	4.13 (5)	0.73
Ischemic heart disease (%)	5.79 (7)	3.31 (4)	0.35
History of heart attack (%)	3.31 (4)	1.65 (2)	0.41
History of ischemic stroke (%)	24.79 (30)	15.7 (19)	0.08
Atrial fibrillation (%)	0 (0)	2.48 (3)	0.08
Lung diseases (%)	3.31 (4)	4.13 (5)	0.73
Hyperthyroidism (%)	3.31 (4)	0.83 (1)	0.18
Hypothyroidism (%)	3.31 (4)	4.13 (5)	0.73
Hypercholesterolemia (%)	9.92 (12)	9.09 (11)	0.83
Current medications			
Acetylsalicylic acid (%)	12.4 (15)	20.66 (25)	0.08
β-blockers (%)	13.22 (16)	18.18 (22)	0.29
Angiotensin-converting enzyme inhibitors (%)	9.92 (12)	20.66 (25)	0.02
AT_2_-blockers (%)	0.83 (1)	0. 83 (1)	0.99
Calcium channel blockers (%)	5.79 (7)	6.61 (8)	0.79
Diuretics (%)	12.40 (15)	9.09 (11)	0.41
Steroids (%)	0.83 (1)	0. 83 (1)	0.99
Antidiabetic therapy (%)	4.96 (6)	2.48 (3)	0.31
Insulin (%)	1.65 (2)	3.31 (4)	0.41
Heparin (%)	0.83 (1)	0. 83 (1)	0.99
Anticoagulants (%)	8.26 (10)	0.83 (1)	< 0.01
Nitrates (%)	1.65 (2)	0 (0)	0.15
Statins (%)	9.92 (12)	4.96 (6)	0.14
Artery sizes			
A1 segment diameter, mm ± SD	1.82 ± 0.46	1.73 ± 0.43	0.12
A2 segment diameter, mm ± SD	1.44 ± 0.47	1.51 ± 0.39	0.18
A3 segment diameter, mm ± SD	1.20 ± 0.36	1.36 ± 0.38	< 0.01
A1–A3 segment length [mm] ± SD	10.70 ± 2.51	9.85 ± 1.68	< 0.01
C7 segment diameter, mm ± SD	3.47 ± 0.72	3.47 ± 0.83	0.98
Tortuosity descriptors			
Relative length ± SD	0.64 ± 0.23	0.56 ± 0.22	< 0.01
Sum of angle metrics ± SD	0.27 ± 0.19	0.18 ± 0.15	< 0.01
Product of angle distance ± SD	0.12 ± 0.13	0.09 ± 0.11	0.02
Triangular index ± SD	0.57 ± 0.14	0.44 ± 0.15	< 0.01
Inflection count metric ± SD	0.10 ± 0.14	0.08 ± 0.09	0.11

**Table 3:** Correlation between aneurysm dome size and tortuosity descriptors

Aneurysm dome size		
Tortuosity descriptor	*R*	*p* value
Relative length	0.116	0.24
Sum of angle metrics	0.089	0.37
Product of angle distance	0.052	0.60
Triangular index	− 0.194	0.047
Inflection count metric	0.094	0.34

## Discussion

We demonstrated that previous myocardial infarction, ischemic stroke, use of anticoagulants, and not being on ACEI are associated with the presence of ACoA aneurysms. In multivariate models, such measures of ACA tortuosity as SOAM, and TI, together with male sex were independently related to the presence of ACoA aneurysms. Greater values of these indices were associated with male sex (SOAM, PAD, TI) and arterial hypertension (SOAM).

Our study showed a significant correlation between higher tortuosity descriptors and certain risk factors of aneurysm development. Coexistence of arterial hypertension and higher vascular tortuosity had been widely described in relation to retinal [2] and coronary [4] vessels, as well as in terms of carotid artery [17], iliac artery [18], and capillary vessels [19]. Likewise, brain white matter arteriole tortuosity was associated with the presence and severity of hypertension [8]. To explain that correlation, it has been shown that the rising of arterial lumen pressure above a certain critical value results in artery instability and might lead to an increase of its tortuosity [20]. Another risk factor related with tortuosity in our study was male gender. Opposite findings were showed in terms of coronary arteries [21] and carotid artery among females older than 60 years old [22]. Previously, we showed that male gender was associated with lower tortuosity of the MCA [15]. However, the fact that ACoA aneurysm development is more common among male patients is consistent with our results [23]. Correlation between SOAM and history of myocardial infarction or ischemic stroke may suggest its influence on blood clot formation. That might result from the fact that higher tortuosity can reduce blood flow velocity, especially in terms of coronary vessels [24]. Another possible explanation may be the post-infarct use of antiplatelet medications. Indeed, we also found the presence of ACoA aneurysms to be higher in patients taking anticoagulants. Also, as mentioned, tortuosity is linked with a higher risk of atherosclerosis [1]. Association of stroke and retinal vessel tortuosity was shown by Ong et al [25]; however, analogous correlation was not shown for carotid artery [22].

We also found that patients with ACoA aneurysm had higher SOAM, PAD, and TI. They also had higher RL. These findings may suggest that local smaller angles in ACA course could impact more on aneurysm development than deviations from a straight line. It is important to note that the natural course of the ACA significantly departs from straight line. Therefore, the RL may not be a suitable tortuosity index of this artery. Higher PAD and TI values for ACoA aneurysm patients may imply that global tortuosity is also higher in arteries of those patients. To the best of our knowledge, only one study analyzed the impact of ACA tortuosity on ACoA aneurysm development; however, the results were inconclusive [26]. Several researchers have demonstrated that a smaller angle between A1 and A2 ACA segments is associated with ACoA aneurysm presence. This is consistent with our finding of higher ACA SOAM in patients with ACoA aneurysm [27, 28]. Labeyrie et al found linkage between cerebral aneurysms development and tortuosity of the internal carotid artery [13]. Similar results for vertebral artery aneurysms were demonstrated by Virgillio et al [14]. In addition, both aortic aneurysm development and risk of its rupture were associated with a higher tortuosity of the aorta [29]. Increased risk of aneurysm formation additionally affects patients who suffer from rare genetic syndromes associated with vessel tortuosity, such as artery tortuosity syndrome and Loeys-Dietz syndrome [30, 31].

An explanation for the association of aneurysm development with higher tortuosity is most likely due to the impairment of the arterial wall. Hemodynamic studies, performed on coronary arteries, showed that higher tortuosity is associated with lower wall shear stress (WSS), prolonged relative residence time (RRT), and disturbed blood flow [32]. Higher RRT promotes endothelial dysfunction and leads to atherosclerosis [32, 33], which weakens the arterial wall and might initiate aneurysm development [34, 35]. Association of cerebral atherosclerosis and higher tortuosity was shown in a study by Kim et al [36]. Lower WSS might also lead to inflammatory response and extracellular matrix degradation [37]. Additionally, as tortuosity itself results from arterial wall remodeling [1], it may imply the involvement of factors that favor this process. As previously mentioned, tortuosity could result from elastin degradation in the arterial wall [12], as well as increased blood flow [38] and blood pressure [11]. Also, the study performed by Zhang et al on rat models showed that arterial buckling, which is a state of arterial instability preceding the development of tortuosity, is associated with higher cell proliferation and increased expression of matrix metalloproteinase-2 [39]. Higher expression of metalloproteinases indicates wall remodeling and is also present in aneurysms [40]. Artery buckling was also showed to reduce endothelial nitric oxide synthase production [41], which could promote aneurysm development as well [42]. This may in part account for the fact that in our study, patients on ACEI had less ACoA aneurysms. A surprising finding of our study was the negative correlation between TI and aneurysm size. According to our knowledge, there are no studies analyzing the impact of cerebral vessel tortuosity on aneurysm growth. However, regarding aortic aneurysms, it was shown that increased tortuosity is associated with a higher risk of its growth [29]. Also, higher tortuosity of the aorta was proved to be related to increased maximum wall stress [43]. WSS seems to be inversely correlated with arterial tortuosity in the cerebral blood vessels [24]. These facts could explain the protective influence of tortuosity on aneurysm growth. However, it was showed that both higher and lower deviations of WSS may influence aneurysm development and rupture, as both of these situations could lead to arterial wall damage [44]. It was also suggested that lower WSS might contribute to larger aneurysm formation. Despite that, it still remains unclear whether decreased WSS is a result of feeding artery shape or an aneurysm itself [44].

An interesting finding of this and our previous study, concerning MCA [15], is the differences in the association of SOAM and PAD with aneurysm presence. As ACoA is a communicating artery, which is supplied by both ICAs, it is characterized by different hemodynamics than MCA. Wang et al [45] study showed that WSS and flow rate in ACoA aneurysm are lower than in MCA aneurysm. Therefore, changes in hemodynamics parameters, resulting from increase or decrease tortuosity, might influence differently these two artery aneurysm formations. However, research concerning that influence, which might in-depth explain the results of our studies, have not been done so far.

In our previous study, we also found that lower tortuosity of ACA is related to higher risk of ACoA aneurysm rupture [46]. It is consistent with our findings concerning aneurysm growth, but opposite to our findings concerning aneurysm presence. These results also require further analysis of hemodynamics in both ACA and ACoA aneurysm. However, they show that changes in blood flow leading to aneurysm development might further act as protective factor against its growth and rupture. Our study needs to be considered in the context of its possible limitations. The study was retrospective, and the relations we demonstrate cannot be viewed as causal. Another major limitation of our study was the inability to measure ACA tortuosity before aneurysm development. Therefore, the impact of aneurysm presence on tortuosity changes still remains unclear. Additionally, our control group consisted of patients with intracranial aneurysm in different locations than ACoA. This was due to the fact that patients without cerebral aneurysms rarely undergo DSA. Despite this, it is the first study that demonstrates an association between ACA tortuosity and risk of ACoA aneurysm development and growth.

## Conclusions

Higher global tortuosity and smaller local angles may contribute to intracerebral aneurysm development, most likely due to the higher risk of arterial wall impairment and further remodeling. However, increased tortuosity, following a decrease in blood flow and wall shear stress, could be a protective factor against aneurysm growth.

## Abbreviations

ACA: Anterior cerebral artery
ACEI: Angiotensin-converting enzyme inhibitors
ACoA: Anterior communicating artery
DSA: Digital subtraction angiography
ICA: Internal carotid artery
ICM: Inflection count metrics
MCA: Middle cerebral artery
PAD: Product of angle distance
RL: Relative length
RRT: Relative residence time
SOAM: Sum of angle metrics
TI: Triangular index
WSS: Wall shear stress
